# Fine mapping of *Pi57*(t) conferring broad spectrum resistance against *Magnaporthe oryzae* in introgression line IL-E1454 derived from *Oryza longistaminata*

**DOI:** 10.1371/journal.pone.0186201

**Published:** 2017-10-10

**Authors:** Liying Dong, Shufang Liu, Peng Xu, Wei Deng, Xundong Li, Didier Tharreau, Jing Li, Jiawu Zhou, Qun Wang, Dayun Tao, Qinzhong Yang

**Affiliations:** 1 Agricultural Environment and Resources Institute, Yunnan Academy of Agricultural Sciences, Kunming, Yunnan, China; 2 Food Crops Institute, Yunnan Academy of Agricultural Sciences, Kunming, Yunnan, China; 3 Key Laboratory of Tropical Plant Resources and Sustainable Use, Xishuangbanna Tropical Botanical Garden, Kunming, Yunnan, China; 4 Centre de Coopération Internationale en Recherche Agronomique pour le Développement, UMR BGPI, Montpellier, France; Fujian Agriculture and Forestry University, CHINA

## Abstract

Wild species of the genus *Oryza* are excellent gene pools for improvement of agronomic traits of Asian cultivated rice. The blast resistance gene *Pi57*(t) in the introgression line IL-E1454 derived from *Oryza longistaminata* was previously mapped on rice chromosome 12. Inoculation with 322 *Magnaporthe oryzae* isolates collected from 6 countries indicated that *Pi57*(t) conferred broad spectrum resistance against *M*. *oryzae*. Two mapping populations consisting of 29070 and 10375 F_2_ plants derived from the crosses of resistant donor IL-E1454 with susceptible parents RD23 and Lijiangxintuanheigu respectively, were used for fine mapping of *Pi57*(t) locus. Based on genotyping and phenotyping results of recombinants screened from the two crosses, *Pi57*(t) was finally mapped to a 51.7-kb region flanked by two molecular markers (STS57-320 and STS57-372) on the short arm and close to the centromere of chromosome 12. Six candidate resistance genes were predicted in the target region according to the reference sequence of Nipponbare. These results could facilitate both marker-assisted selection for disease-resistant breeding and gene cloning.

## Introduction

Rice blast, caused by the filamentous ascomycete *Magnaporthe oryzae* [1], is one of the most destructive diseases for rice (*Oryza sativa* L.), and is responsible for significant yield losses under favorable environmental conditions worldwide [2]. Rice-*M*. *oryzae* pathosystem follows the gene-for-gene relationship during the host-pathogen interaction [3–4]. The use of resistance (*R*) genes in rice breeding has been proved to be the most economic, effective and environment-friendly strategy for blast management. But, after the release of blast resistant varieties, the emergence of virulent races of the pathogen often cause the rapid loss of effectiveness of resistance conferred by monogenic resistance [5]. Few exceptions of monogenic durable resistance exist [6]. Monogenic resistances may also contribute to durable resistance if appropriate management strategies are used: agronomic conditions, rotation and/or mixtures of varieties, etc. [7–10]. In addition, the pyramiding of multiple *R* genes with different resistance spectra to races of *M*. *oryzae* into a single variety through marker-assisted selection strategy is one of the most effective methods to breed durable varieties for durable control [11]. Pyramiding requires the characterization and identification of markers closely linked to the *R* genes of interest.

In the past decades, genetic studies on blast resistance in rice have been extensively conducted, and over 100 major blast *R* genes from *O*. *sativa* and its wild relatives have been identified and mapped on the 12 chromosomes of rice [12–16]. Clusters of functional genes were identified on chromosomes 6, 11 and 12. Most of the *R* genes are dominant, except 3 recessive genes, *pi21*[17], *pi55*(t) [18] and *pi66*(t) [16]. The availability of rice genome sequences of two subspecies of cultivated rice, *O*. *sativa* ssp. *japonica* cultivar Nipponbare [19] and *indica* cultivar 9311 [20], greatly facilitate the development of molecular markers for fine mapping of *R* genes, and comparison of *R* gene positions between mapping populations.

Blast *R* gene *Pi57*(t) is carried by a introgression line IL-E1454, and was introgressed from *O*. *longistaminata* into *indica* cultivar RD23. Previously, this gene was preliminary mapped on chromosome 12 of rice using a BC_4_F_2_ population derived from the cross between IL-E1454 and the recurrent parent RD23. *Pi57*(t) was mapped to a 6.07 Mb region between molecular marker RM27892 and RM28093 [21]. Although *Pi57*(t) can be differentiated from known *R* genes *Pita*, *Pita2*, *Pi12*, *Pi19* and *Pi20* also located on chromosome 12, through pathogen-testing with different *M*. *oryzae* isolates [21], its exact genomic position on chromosome 12 remains unclear. In this study, two mapping populations from ILE1454/RD23 and IL-E1454/Lijiangxintuanheigu (LTH) were used for further mapping of *Pi57*(t).

## Materials and methods

### Mapping population construction, planting and resistance evaluation

Resistant donor parent IL-E1454 was crossed with susceptible cultivars RD23 (*indica*) and LTH (*japonica*), respectively. The IL-E1454/RD23 and IL-E1454/LTH F_1_ plants were grown in the greenhouse to generate F_2_ populations for gene mapping. The germinated F_2_ seeds of the IL-E1454/RD23 and IL-E1454/LTH cross combinations were sown in trays of 20×12×5 cm filled with compost, and each tray sowed with 95 seeds. Seedlings were inoculated with *M*. *oryzae* strain HN09-1C-7 by spraying at 4 leaf stages with 20 ml conidial suspension per tray. The inoculated rice plants were stored for one night in a controlled dark chamber at 25°C with 95% relative humidity, and then transferred back to the greenhouse. Lesion types on rice leaves were observed 6–7 days after inoculation and scored according to a standard reference scale [3]. Plants scored from 1 to 3 were considered to be resistant and scored from 4 to 6 were considered to be susceptible. Four hundred and seventy-five seedlings and 570 of the IL-E1454/RD23 and IL-E1454/LTH populations respectively were inoculated and evaluated for the expected 3:1 resistant: susceptible segregation ratio in F_2_ populations [21]. To determine the resistance spectrum of *Pi57*(t) locus, IL-E1454 and 10 monogenic lines were inoculated with 322 isolates from 6 countries (S1 Table).

### *M*. *oryzae* isolate cultivation

*M*. *oryzae* isolate HN09-1C-7, virulent to RD23 and LTH but avirulent to IL-E1454, and previously used to map *Pi57*(t) [21] was cultured on oatmeal medium (20 g of oatmeal, 15 g of agar, 10 g of sucrose and 1 L of distilled water) for 7 days in dark incubator at 25°C, and then aerial mycelia were washed off by gentle rubbing with distilled water and paintbrush. The colonies were then successively exposed to fluorescent light for 3 days to induce sporulation at 25°C. Conidia were harvested by softly scraping and flooding the medium surface with distilled water containing 0.01% Tween 20 detergent. The concentration of conidial suspension was adjusted to 50000 conidia/ml for inoculation.

### Marker development and genetic map construction

Total DNA was extracted from fresh leaves of each plant following the method of Edwards et al. [22]. The SSR markers located in the genomic region carrying *Pi57*(t) and producing a polymorphic band between parents were used to genotype the mapping population. Sequence-tagged site (STS) markers were developed based on the alignment (using BLAST) within the critical region of the genomic sequences of 93–11 and Nipponbare.

PCR amplification conditions consisted of a denaturing step of 94°C/3 min, followed by 35 cycles of 94°C/30 s, annealing temperature (see Table 1)/30 s, and 72°C/1 min, ending with an extension step of 72°C/7 min. Amplicons were separated by 8% polyacrylamide gel electrophoresis and visualised by silver staining. Primer sequences and other relevant properties of the marker assays are summarized in Table 1. The polymorphism determined by all STS markers developed in this study among resistant donor IL-E1454, and susceptible parents RD23 and LTH were showed in S1 Fig. The genetic and linkage map of polymorphic markers was constructed using MAPMAKER/EXP 3.0 [23]. The Kosambi mapping function was used to transform recombination frequency to genetic distance (cM).

**Table 1:** Summary of PCR markers used in this study.

Marker	Primer sequence (5'-3')^a^	Genomic position (bp)^b^	Anneling temperature (°C)	Expected size (bp)
RM27892	F: ATAAGAGATGGCCGCTTGAGAGC	9504613–9504635	55	153
	R: GTGACACATGGTGACTCGAGAGC	9504765–9504743		
RM27921	F: CTTCCTCCTCCTCTCCTTCTTCC	10196011–10196033	55	199
	R: GAAGCTCTTCTACTTGCCGTTCC	10196209–10196187		
RM7102	F: TAGGAGTGTTTAGAGTGCCA	13214191–13214172	55	168
	R: TCGGTTTGCTTATACATCAG	13214024–13214043		
RM28093	F: CTGTTTAGGAGCGTTTGTAGG	15572389–15572409	55	113
	R: ATTAAGTCACGGCCTGTCAC	15572502–15572483		
STS57-1	F: TGGATGAAGAAATGTTACCCAA	10467998–10467977	55	105
	R: GAAGAATGCAGGTCACAGACA	10467894–10467914		
STS57-44	F: TAGAATTACGACAGGAAAAAC	10742010–10742030	55	81
	R: CACAACCCTTGAAAAAAAGC	10742090–10742071		
STS57-36	F: CTAACCAGGACCTATAACCAG	10770604–10770624	55	96
	R: GTCACTGATGGTCATACTATTG	10770699–10770678		
STS57-320	F: GAGGTGGAGGTGGAGGTCGATAGA	10799295–10799318	60	85
	R: ATCACCATCCATTCTTACCAGTTTTC	10799379–10799354		
STS57-336	F: TCCACCGAGCAAAAACCT	10804274–10804291	55	102
	R: GACGGCGATCTGGGGCTGCTC	10804375–10804355		
STS57-4	F: CCCAACGCGTGTTGTATCTCTTGA	10833471–10833494	60	253
	R: GAAATGGAGCAGTACCGTATAGGC	10833723–10833700		
STS57-372	F: TGTAGAATATGTGCACATGA	10850853–10850872	55	106
	R: CTGCATGGAAAAAATATGTG	10850958–10850939		
STS57-72	F: TGCCAGGAATGCATAGTGGA	10905413–10905432	55	95
	R: CAGCTATGACTCCGTGACCTC	10905507–10905487		
STS57-2	F: CGAATTTCTATACTACCTCTGTTCC	11367028–11367052	55	231
	R: GCAAGGATAAACAAATCATGTAGC	11367258–11367235		
18690^c^	F: ATGGGAGGCTTCAGTCTTCATCG	10799671–10799693	65	2528
	R: TCAAGAGATACAACACTCGTTGGGAT	10802198–10802173		
18700^c^	F: TCACTCCTCTTCCTCTACCCGCGAAG	10807162–10807187	65	1861
	R: ATGCCCTCCACGCCCACATCC	10809022–10809002		
18710^c^	F: ACGGCCATGACAAGTTGTCGTAAGA	10815080–10815104	65	1792
	R: TGGCCCTCTCCTCTCTCCCCTACAA	10816871–10816847		
18729–1^C^	F: ATGGACAGGCTCTGGGCGGCTCCT	10822943–10822966	65	1905
	R: CTCTAATGCATGCTTGTTAACTAGTTG	10824847–10824821		
18729–2^d^	F: TCATGGTCATATGTTGCAAGACAAAT	10824782–10824807	65	8713
	R: TCAAGAGATACAACACGCGTTGGGA	10833494–10833470		
18750^c^	F: ATGGGCCTCATGCACGCACTCCTC	10843399–10843376	68	1863
	R: CAAGCCCTATCGATGTAATACTGTT	10841537–10841562		
18760^d^	F: AACGGTGGGAGCCTTGGGAGT	10848266–10848286	68	7960
	R: CAAACCAGGCTCCGACAGCGAA	10856226–10856205		

^a^ F forward, R reverse

^b^ Genomic position of each marker along chromosome 12 as determined by BLASTN analysis against the Nipponbare genome sequence (IRGSP 1.0)

^c^ The PCR were performed as following: after preheating for 1 min at 98°C, 35 PCR cycles (10 s at 98°C, 30 s at 65°C, and 3 min at 68°C), followed by 7 min at 72°C, the PCR products were analyzed by 1% agarose gel

^d^ The PCR were conducted as following: after preheating for 1 min at 98°C, 35 PCR cycles (10 s at 98°C, 30 s at 65°C, and 10 min at 68°C), followed by 10 min at 72°C, the PCR products were analyzed by 0.8% agarose gel

### Physical map construction *in silico* and candidate gene prediction

To construct a physical map of *Pi57*(t) locus, all molecular markers used for gene mapping were landed on the IRGSP1.0 pseudomolecule of reference cv. Nipponbare released by IRGSP through BLASTN search (https://www.ncbi.nlm.nih.gov/Blast.cgi). Subsequently, the physical map spanning *Pi57*(t) locus was constructed based on the reference genomic sequence of Nipponbare. The candidate *R* genes in the target region were predicted based on the annotation information by Rice Genome Annotation Project (http://rice.plantbiology.msu.edu/), GENSCAN (http://genes.mit.edu) and FGENSH (http://www.softberry.com/) software.

### Amplification of candidate *R* genes from IL-E1454 by PCR

To amplify the candidate *R* genes from IL-E1454, PCR primers were designed according to the reference genomic sequence of Nipponbare. The long-range enzyme (PrimeSTAR GXL DNA polymerase, TAKARA BIO INC.) was used to amplify the target DNA fragments. The PCR amplification conditions and primers information were summarized in Table 1. After amplification, the PCR products were then sequenced and analyzed.

## Results

### Genetic analysis of *Pi57*(t) locus

Altogether, 475 and 570 F_2_ plants derived from the crosses of IL-E1454/RD23 and IL-E1454/LTH, were inoculated with blast isolate HN-09-1C-7 for genetic analysis, respectively. As a result, phenotype of resistant (R) and susceptible (S) plants in both these two populations fitted the expected segregation ratio of 3:1, i.e. 360 R: 115 S (*χ*^2^ = 0.1579, *P* = 0.69), and 437 R: 133 S (*χ*^2^ = 0.8444, *P* = 0.35), respectively. Hence, a single dominant gene in IL-E1454 confers resistance to *M*. *oryzae* isolate HN-09-1C-7, confirming previous results [21]. Therefore, both populations were used to finely map *Pi57*(t) locus.

### Fine mapping of *Pi57*(t) locus

**Fig 1:**
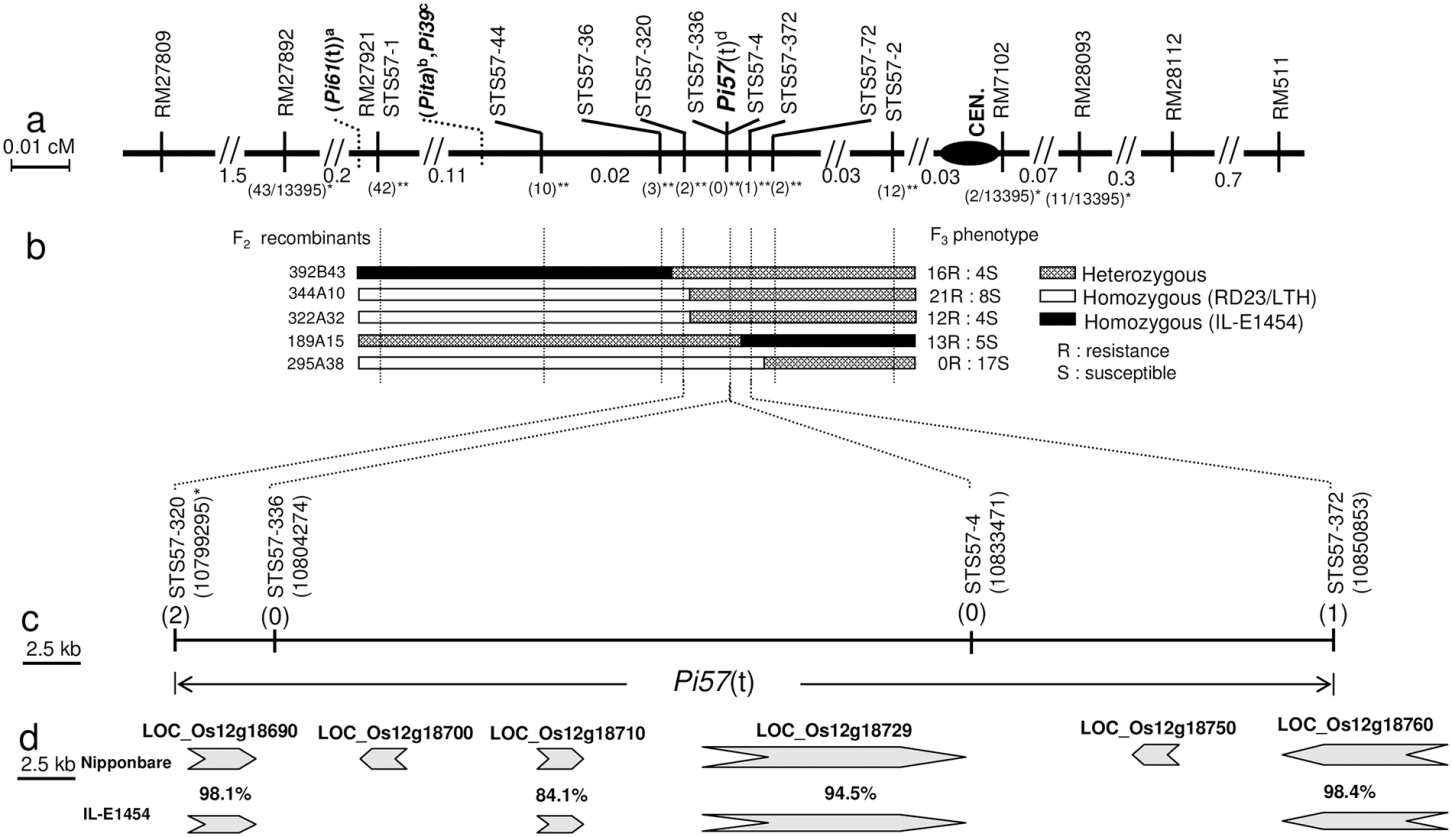
Genetic and physical maps of the region covering the *Pi57*(t) locus. **a** An integrated genetic map of rice chromosome 12, including 3 finely mapped *R* genes and cloned *Pita* gene. Map positions were inferred from a: *Pi61*(t) [13]; b: *Pita* [24]; c: *Pi39*(t) [25]; d: *Pi57*(t) (this study). *: recombinants/population size; **: recombinants screened from 39445 F_2_ individuals; CEN. Centromere; Map distances in cM. **b** Recombinants and their phenotypes delimited by molecular markers STS57-36 and STS57-72. **c** Physical map of the *Pi57*(t) locus based on Nipponbare genome sequence. *: represent the chromosomal position of molecular markers on Nipponbare genomic sequence of chromosome 12, the numbers in parentheses under the molecular markers represent the number of recombinants between *Pi57*(t) and the marker locus. **d** The predicted candidate *R* genes for *Pi57*(t) in both IL-E1454 and Nipponbare.

In a previous study, *Pi57*(t) gene were preliminary mapped in a region spanning the centromere of chromosome 12, and delimited between two SSR markers (RM27892 and RM28093). To further map its chromosomal position, 13395 F_2_ plants from IL-E1454/RD23 were genotyped by RM27892 and RM28093. As a result, 54 recombinants were found between markers RM27892 and RM28093. The recombinants were further genotyped with 2 known SSR markers RM27921, RM7102, and 3 new developed STS markers STS57-1, STS57-2 and STS57-4. The results showed that recombination events at RM27892, RM27921, STS57-1, STS57-4, STS57-2, RM7102 and RM28093 were 43, 16, 16, 0, 2, 2 and 11, respectively (Fig 1a). Based on the genomic positions of the molecular markers, *Pi57*(t) locus was delimited between STS57-1 and STS57-2 (Fig 1a), and co-segregates with STS57-4. In order to finely narrow down the region carrying *Pi57*(t) locus, 15675 and 10375 additional F_2_ plants from the crosses of IL-E1454/RD23 and IL-E1454/LTH, respectively, were genotyped with STS57-1 and STS57-2. Altogether, 42 and 12 recombinants were identified at STS57-1 and STS57-2 (Fig 1a). Then, these recombinants were further genotyped with STS57-4 and 3 new developed polymorphic STS markers STS57-44, STS57-36 and STS57-72, which are located between STS57-1 and STS57-2. As showed in Fig 1a, 10 and 3 recombinants were found between STS57-44 or STS57-36 and STS57-1, respectively. Two recombinants were found between STS57-72 and STS57-2. Through phenotype assays of the recombinants, the *Pi57*(t) locus was further mapped in the region of STS57-36 and STS57-72, and co-segregates with STS57-4 (Fig 1a).

Three additional STS markers (STS57-320, STS57-336 and STS57-372), were developed in the STS57-36/STS57-72 interval. Subsequently, the 54 recombinants between STS57-1 and STS57-2 were genotyped with these markers. As showed in Fig 1a and 1b, two recombinants were detected between STS57-320 and STS57-36, and only 1 recombinant was identified between STS57-372 and STS57-72. As a consequence of fine mapping, *Pi57*(t) gene was finally narrowed down to the region between STS57-320 and STS57-372, and co-segregates with STS57-4 and STS57-336 (Fig 1a–1c).

### Construction of physical map of *Pi57*(t) locus, candidate gene prediction and amplification

All the molecular markers closely linked with *Pi57*(t) gene were landed to the genome sequence of chromosome 12 of reference cultivar Nipponbare by BLAST analysis (Fig 1c), and the phenotypes of recombinants between STS57-320 and STS57-372 were showed in Fig 1b. Subsequently, *Pi57*(t) locus defined by two flanking markers (STS57-320 and STS57-372), and co-segregated with two markers (STS57-336 and STS57-4). The resulting physical map is shown in Fig 1c with a physical distance of ca. 51.7 kb in the target region.

Based on the annotated Nipponbare genome sequence, 6 genes (*LOC_os12g18690*, *LOC_os12g18700*, *LOC_os12g18710*, *LOC_os12g18729*, *LOC_os12g18750*, and *LOC_os12g18760*) were predicted in the target region flanked by STS57-320 and STS57-372 (Chr12:10799294 to10850958). Among these candidate genes, all genes encode uncharacterized protein with the exception of *LOC_os12g18760* that encode a putative peptidase family C78 domain containing protein. When amplifying these candidate genes in IL-E1454 using primers designed based on the genome sequence of Nipponbare, the candidate genes *LOC_os12g18690*, *LOC_os12g18710*, *LOC_os12g18729*, and *LOC_os12g18760* were successfully obtained, and showed high homology to the corresponding gene loci in Nipponbare at a level of 98.1%, 84.1%, 94.5% and 98.4%, respectively (Fig 1d). The candidate gene *LOC_os12g18700* and *LOC_os12g18750* could not be amplified. When new PCR primers were designed to amplify the remaining target region with IL-E1454 DNA as the template, two gaps located between *LOC_os12g18690*/*LOC_os12g1871*, and *LOC_os12g18729*/*LOC_os12g18760* could not be successfully filled out (data not shown), these suggested that *LOC_os12g18700* and *LOC_os12g18750* genes would be absent or inserted with large DNA fragments in the gaps region, respectively.

### Resistance spectrum determination of *Pi57*(t) gene

To determine the resistance spectrum, identity and potential usefulness of *Pi57*(t) in rice breeding for disease resistance, IL-E1454 and 10 monogenic lines were tested with 322 *M*. *oryzae* isolates from Cambodia, Laos, Myanmar, Thailand, Vietnam and China (Table 2). The inoculation results showed that IL-E1454 was resistant to 300 isolates (93.17%) of the total tested isolates. Compared with monogenic lines carrying *R* genes located on chromosome 12, IL-E1454 was resistant to all isolates from Laos and Myanmar, indicated that *Pi57*(t) could be differentiated from *Pi12*, *Pi19*, *Pi20*, *Pita* and *Pita-2*, due to their susceptibility to part of the isolates from Laos and/or Myanmar. Meanwhile, *Pi57*(t) showed a high resistant frequency to the isolates used in this study with the comparison of the known broad-spectrum *R* genes (*Pi5*, *Piz*, *Piz-5*, *Piz-t* and *Pi9*), suggesting that *Pi57*(t) gene conferred a broad spectrum resistance against *M*. *oryzae*.

**Table 2:** Comparison of resistant percentage of IL-E1454 and 10 monogenic lines to 322 *Magnaporthe oryzae* from 6 countries.

Number of *M*. *oryzae* isolates	Country of origin	Lines											
		IL-E1454 (*Pi57*(t))	IRBL12-M (*Pi12*)^a^	IRBL19-A (*Pi19*)	IRBL20-IR24 (*Pi20*)	IRBLTA-K1 (*Pita*)	IRBLTA2-PI(*Pita-2*)	IRBL5-M *(Pi5*)	IRBLZ FU (*Piz*)	IRBLZ5-CA (*Piz-5*)	IRBLZT-T (*Piz-t*)	IRBL9-W (*Pi9*)	LTH
44	Cambodia	95.45^b^	50.00	9.09	50.00	79.55	100.00	97.73	54.55	47.73	18.18	100.00	0
30	Laos	100.00	43.33	80.00	86.67	56.67	6.67	70.00	66.67	70.00	100.00	100.00	0
25	Myanmar	100.00	40.00	20.00	76.00	64.00	100.00	100.00	96.00	96.00	12.00	96.00	0
19	Thailand	83.33	78.95	26.32	21.05	100.00	100.00	100.00	94.74	89.47	36.84	100.00	0
28	Vietnam	78.57	89.29	21.43	21.43	85.71	75.00	89.29	64.29	57.14	82.14	100.00	0
176	China	90.91	81.25	20.45	69.32	58.52	68.18	96.02	55.68	51.14	68.18	97.16	0

^a^ Resistant gene carrying in monogenic line;

^b^ Resistance percentage(%), Number of isolates avirulent to a line or *R* gene/total number of isolates tested×100

## Discussion

In a previous study, *Pi57*(t) was identified and preliminary mapped in 6.07 Mb region on chromosome 12 of rice [21]. In this study, this *O*. *longistaminata*-derived gene was finely mapped to a region of 51.7 kb on the short arm proximal to centromeric position of chromosome 12 of rice, by using two mapping population from IL-E1454/RD23 and IL-E1454/LTH. It has been well documented that the recombination frequency along a chromosome is quite different in plant, and that the chromosomal recombination was significantly suppressed in the region with more repetitive DNA sequences and/or close to the centromeric regions than other regions [26–28]. *Pi57*(t) was located in the region close to centromere of chromosome 12 and we observed low recombination frequency: after mapping with 13395 F_2_ individuals, *Pi57*(t) locus was still mapped in a large chromosomal region flanked by molecular markers STS57-1 and STS57-2. Although the population size used in this study are relatively larger than those used in other genes mapping [13, 25], increasing mapping population consisting of 39445 F_2_ individuals could finally delimit this locus to an estimated 51.7 kb, based on the physical distance determined by in silico mapping on *O*. *sativa* reference genome.

Classical genetics and molecular data have demonstrated that many resistance genes in plant are often clustered in a certain chromosomal region as a complex locus [29]. To date, 19 *R* genes *Pita*, *Pita2*, *Pitq6*, *PiGD-3*, *Pi6*(t), *Pi12*(t), *Pi19*(t), *Pi20*(t), *Pi21*(t), *Pi24*(t), *Pi31*(t), *Pi32*(t), *Pi39*(t), *Pi41*, *Pi42*(t), *Pi57*(t), *Pi58*(t), *Pi61*(t) and *Pi157*(t) have been mapped on chromosome 12, and most of them are concentrated around the centromere region [12, 13, 21, 30, 31–34]. Most of them were mapped to a relative large chromosomal region spanning over several Mb on the short arm of chromosome 12 [12, 31, 33–34]. By using a large number of *M*. *oryzae* strains, *Pi57*(t) could be differentiated from genes located in the same genomic regions and introgressed in monogenic lines (*Pita*, *Pita-2*, *Pi12*, *Pi19* and/or *Pi20*). However, the positional or allelism relationship among these genes could not be compared with each other in detail, due to the limited information about their rough mapping position and different *M*. *oryzae* strains used in gene mapping research [13, 21, 31, 33–34]. Fine mapping of *R* genes provide direct information about the relationship among the genes in a cluster. For example, by comparing with the chromosomal position of cloned *Pita* gene, *Pi61*(t) gene was mapped at ca. 200 kb region on the telomere side, and *Pi39*(t) was localized at 37 kb region on the centromeric side in the short arm of chromosome 12 [13, 24–25]. *Pi57*(t) was mapped to the proximal side to the centromere compared with *Pi39*(t) location, indicating that this is a new locus conferring resistance to rice blast. With the characterization of broad spectrum resistance against *M*. *oryzae*, this gene would be a very useful gene resource for improvement of resistance to rice blast in rice breeding program.

Most of the *R* genes cloned from plants so far encode protein with nucleotide-binding site and leucine rice repeat (NBS-LRR), LRR-kinase or kinase structure [35]. To date, all cloned rice blast *R* genes encode NBS-LRR proteins, except for *Pid-2* and the recessive *pi21*, which encode a receptor-like kinase protein and a proline-rich protein, respectively[36, 37]. In this study, *Pi57*(t) gene was mapped in a region containing 6 predicted genes without any similarity to known *R* genes, based on the gene annotation results of reference genomic sequence of *O*. *sativa* ssp. *japonica* cultivar Nipponbare. In the present study, because two gaps exit in target region in IL-E1454 with the comparison of Nipponbare genomic sequence, whether *Pi57*(t) encodes protein with either novel structure similar to the annotated candidate gene, or a known *R* gene structure but located in gap region in IL-E1454 remains to be clarified. Currently, the gap-filling with genome walking strategy and genetic transformation for candidate genes are undergoing.

## Supporting information

S1 Fig Polymorphic analysis of resistance donor IL-E1454, and susceptible parents RD23 and LTH with STS markers developed in this study. (PDF)

S1 Table Resistance reaction of IL-E1454 and 10 monogenic lines to 322 *Magnaporthe oryzae* strains. (DOC)
