# Supplementary material for: Fine mapping of Pi57(t) conferring broad spectrum resistance against Magnaporthe oryzae in introgression line IL-E1454 derived from Oryza longistaminata
Source: PLoS One. 2017 Oct 10;12(10):e0186201. doi: 10.1371/journal.pone.0186201 (PMC5634632; doi:10.1371/journal.pone.0186201)
Supplement: S1 Fig — (PDF) [file pone.0186201.s001.pdf]

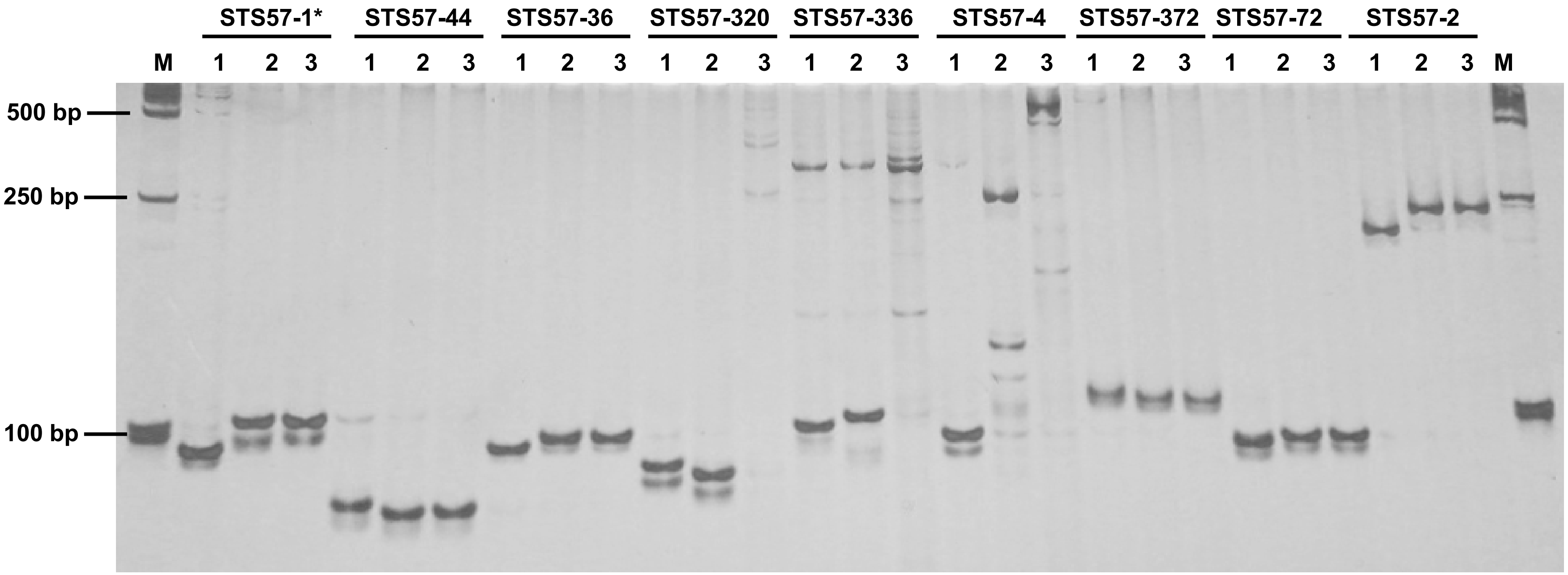

S1 Fig. Polymorphic analysis of resistance donor IL-E1454, and susceptible parents RD23 and LTH with STS markers developed in this study

M: DNA marker, DL2000; *: STS marker; 1=IL-E1454; 2=RD23; 3=LTH. No target PCR fragments were amplified in STS57-320 and STS57-336 loci with LTH genomic DNA as template.
