# Supplementary material for: Fine mapping of Pi57(t) conferring broad spectrum resistance against Magnaporthe oryzae in introgression line IL-E1454 derived from Oryza longistaminata
Source: PLoS One. 2017 Oct 10;12(10):e0186201. doi: 10.1371/journal.pone.0186201 (PMC5634632; doi:10.1371/journal.pone.0186201)
Supplement: S1 Table — (DOC) [file pone.0186201.s002.doc]

| S1 Table Resistance reaction of IL-E1454 and 10 monogenic lines to 322 *Magnaporthe oryzae* strains | | | | | | | | | | | | | |
| --- | --- | --- | --- | --- | --- | --- | --- | --- | --- | --- | --- | --- | --- |
| Strains of *Magnaporthe oryzae* | Origin | **IL-E1454 (*Pi57*(t))** | IRBL12-M (*Pi12*) | IRBL19-A (*Pi19*) | IRBL20-IR24 (*Pi20*) | IRBLTA-K1 (*Pita*) | IRBLTA2-PI(*Pita-2*) | IRBL5-M *(Pi5*) | IRBLZ FU (*Piz*) | IRBLZ5-CA (*Piz-5*) | IRBLZT-T (*Piz-t*) | IRBL9-W (*Pi9*) | LTH |
|
| C1-45-1 | Cambodia | R | R | S | R | R | R | R | S | S | R | R | S |
| C2-9-1 | Cambodia | R | S | S | S | R | R | R | S | S | S | R | S |
| C3-10-1 | Cambodia | R | R | S | S | R | R | R | S | S | S | R | S |
| C3-1-2 | Cambodia | S | S | S | S | R | R | R | R | S | S | R | S |
| C3-12-1 | Cambodia | R | S | S | R | R | R | R | R | S | S | R | S |
| C3-13-3 | Cambodia | R | S | S | S | R | R | R | R | R | S | R | S |
| C3-14-1 | Cambodia | R | R | R | R | R | R | R | R | R | R | R | S |
| C3-15-2 | Cambodia | R | S | S | R | R | R | R | R | R | S | R | S |
| C3-16-1 | Cambodia | R | R | S | R | R | R | R | R | R | R | R | S |
| C3-17-1 | Cambodia | R | S | S | S | R | R | R | S | S | R | R | S |
| C3-18-1 | Cambodia | R | R | R | S | R | R | R | R | R | S | R | S |
| C3-20-2 | Cambodia | R | S | S | S | R | R | R | S | S | S | R | S |
| C3-21-2 | Cambodia | R | S | S | R | R | R | R | R | R | R | R | S |
| C3-2-2 | Cambodia | S | S | S | S | R | R | R | S | S | S | R | S |
| C3-22-1 | Cambodia | R | R | S | S | R | R | R | S | S | S | R | S |
| C3-23-3 | Cambodia | R | S | S | S | S | R | R | R | R | S | R | S |
| C3-26-2 | Cambodia | R | S | S | S | R | R | R | S | S | S | R | S |
| C3-3-1 | Cambodia | R | S | S | R | R | R | R | S | S | S | R | S |
| C3-5-1 | Cambodia | R | R | S | S | R | R | R | R | S | S | R | S |
| C3-6-1 | Cambodia | R | S | S | S | R | R | R | S | S | S | R | S |
| C3-7-2 | Cambodia | R | S | S | S | R | R | R | S | S | S | R | S |
| C3-8-1 | Cambodia | R | S | S | S | R | R | R | S | S | S | R | S |
| C3-9-2 | Cambodia | R | R | S | R | R | R | R | R | R | R | R | S |
| C5-10-1 | Cambodia | R | S | S | S | S | R | R | R | R | S | R | S |
| C5-1-1 | Cambodia | R | S | S | S | R | R | R | S | S | S | R | S |
| C5-11-2 | Cambodia | R | S | S | R | R | R | R | R | R | S | R | S |
| C5-12-2 | Cambodia | R | R | S | R | R | R | R | S | S | S | R | S |
| C5-13-1 | Cambodia | R | R | S | R | R | R | R | R | R | S | R | S |
| C5-14-1 | Cambodia | R | R | S | R | S | R | R | R | R | S | R | S |
| C5-15-1 | Cambodia | R | S | S | S | R | R | R | R | R | S | R | S |
| C5-16-1 | Cambodia | R | S | S | S | R | R | R | R | R | R | R | S |
| C5-17-1 | Cambodia | R | S | S | S | R | R | R | R | R | S | R | S |
| C5-18-3 | Cambodia | R | R | S | R | S | R | R | R | R | S | R | S |
| C5-19-1 | Cambodia | R | R | S | R | R | R | S | R | R | S | R | S |
| C5-20-1 | Cambodia | R | R | R | R | R | R | R | R | R | S | R | S |
| C5-21-1 | Cambodia | R | R | S | R | S | R | R | S | S | S | R | S |
| C5-2-2 | Cambodia | R | S | S | S | R | R | R | S | S | S | R | S |
| C5-3-1 | Cambodia | R | R | S | R | S | R | R | S | S | S | R | S |
| C5-4-1 | Cambodia | R | R | S | R | S | R | R | S | S | S | R | S |
| C5-5-1 | Cambodia | R | R | S | R | S | R | R | S | S | S | R | S |
| C5-6-2 | Cambodia | R | R | S | R | S | R | R | S | S | S | R | S |
| C5-7-1 | Cambodia | R | R | S | S | R | R | R | R | R | S | R | S |
| C5-8-2 | Cambodia | R | R | R | R | R | R | R | R | R | R | R | S |
| C5-9-2 | Cambodia | R | R | S | R | R | R | R | R | R | S | R | S |
| L4-5-9-1 | Lao | R | S | R | R | R | S | R | R | R | R | R | S |
| L4-6-10-1 | Lao | R | R | R | R | S | S | S | R | R | R | R | S |
| L4-6-1-1 | Lao | R | R | R | R | R | R | R | R | R | R | R | S |
| L4-6-16-1 | Lao | R | S | R | R | R | S | S | S | R | R | R | S |
| L4-6-19-1 | Lao | R | S | S | R | R | S | R | R | R | R | R | S |
| L4-6-23-1 | Lao | R | S | R | R | R | R | R | R | R | R | R | S |
| L4-6-24-2 | Lao | R | S | R | R | R | S | S | R | R | R | R | S |
| L4-6-25-1 | Lao | R | S | R | R | S | S | R | S | S | R | R | S |
| L4-6-5-1 | Lao | R | S | R | R | R | S | R | S | S | R | R | S |
| L4-6-7-1 | Lao | R | R | R | R | R | S | R | R | S | R | R | S |
| L4-6-9-1 | Lao | R | R | R | R | R | S | R | S | R | R | R | S |
| L4-7-1-1 | Lao | R | R | R | R | R | S | R | R | R | R | R | S |
| L4-7-11-1 | Lao | R | R | R | R | S | S | S | R | R | R | R | S |
| L4-7-12-1 | Lao | R | R | R | R | R | S | S | R | R | R | R | S |
| L4-7-13-1 | Lao | R | R | R | S | S | S | R | R | R | R | R | S |
| L4-7-15-1 | Lao | R | S | R | R | R | S | R | S | S | R | R | S |
| L4-7-16-1 | Lao | R | S | S | R | S | S | R | S | S | R | R | S |
| L4-7-17-1 | Lao | R | S | R | R | R | S | R | R | S | R | R | S |
| L4-7-2-1 | Lao | R | R | S | R | S | S | R | R | R | R | R | S |
| L4-7-21-1 | Lao | R | S | R | S | S | S | R | R | R | R | R | S |
| L4-7-24-1 | Lao | R | S | R | R | R | S | R | R | R | R | R | S |
| L4-7-4-2 | Lao | R | S | R | R | S | S | R | S | S | R | R | S |
| L4-7-7-1 | Lao | R | S | R | R | R | S | S | R | R | R | R | S |
| L4-7-8-1 | Lao | R | S | R | R | R | S | S | R | R | R | R | S |
| L4-7-9-1 | Lao | R | R | S | R | S | S | R | R | R | R | R | S |
| L5-2-1-1 | Lao | R | S | R | R | S | S | S | R | R | R | R | S |
| L5-2-11-1 | Lao | R | R | S | S | S | S | S | S | S | R | R | S |
| L5-2-3-1 | Lao | R | R | S | S | S | S | R | S | R | R | R | S |
| L5-2-5-1 | Lao | R | R | R | R | R | S | R | R | R | R | R | S |
| L5-2-9-1 | Lao | R | S | R | R | S | S | R | S | S | R | R | S |
| M1-28-1 | Myanmar | R | R | S | S | R | R | R | R | R | S | R | S |
| M1-29-1 | Myanmar | R | R | S | R | R | R | R | R | R | R | R | S |
| M1-30-1 | Myanmar | R | S | S | R | R | R | R | R | R | S | R | S |
| M13-17-1 | Myanmar | R | S | S | R | S | R | R | R | R | S | S | S |
| M3-1-1 | Myanmar | R | R | R | R | R | R | R | R | R | S | R | S |
| M3-13-1 | Myanmar | R | R | S | R | R | R | R | R | R | S | R | S |
| M3-25-1 | Myanmar | R | S | R | R | R | R | R | R | R | S | R | S |
| M3-27-1 | Myanmar | R | R | R | R | R | R | R | R | R | R | R | S |
| M3-3-1 | Myanmar | R | S | S | R | R | R | R | R | R | S | R | S |
| M3-5-1 | Myanmar | R | S | S | R | R | R | R | R | R | S | R | S |
| M3-7-1 | Myanmar | R | R | S | R | R | R | R | R | R | S | R | S |
| M3-8-1 | Myanmar | R | S | S | R | R | R | R | R | R | S | R | S |
| M3-9-1 | Myanmar | R | S | S | R | S | R | R | R | R | S | R | S |
| M5-8-1 | Myanmar | R | S | S | R | R | R | R | R | R | S | R | S |
| M6-10-1 | Myanmar | R | S | S | R | S | R | R | R | R | S | R | S |
| M6-20-1 | Myanmar | R | R | S | S | S | R | R | R | R | S | R | S |
| M6-2-1 | Myanmar | R | S | S | R | S | R | R | R | R | S | R | S |
| M6-21-1 | Myanmar | R | R | S | S | S | R | R | R | R | S | R | S |
| M6-22-1 | Myanmar | R | R | S | S | S | R | R | R | R | S | R | S |
| M6-23-1 | Myanmar | R | S | S | R | R | R | R | R | R | S | R | S |
| M6-24-1 | Myanmar | R | R | R | R | R | R | R | R | R | S | R | S |
| M6-26-1 | Myanmar | R | S | S | S | S | R | R | R | R | S | R | S |
| M6-36-1 | Myanmar | R | S | S | R | S | R | R | S | S | S | R | S |
| M9-6-1 | Myanmar | R | S | R | R | R | R | R | R | R | S | R | S |
| M9-9-1 | Myanmar | R | S | S | S | R | R | R | R | R | R | R | S |
| T1-1-1 | Thailand | R | R | S | S | R | R | R | R | R | S | R | S |
| T2-2-1 | Thailand | R | S | S | S | R | R | R | R | R | S | R | S |
| T3-1-2 | Thailand | R | R | S | S | R | R | R | R | R | S | R | S |
| T1-4-2-3 | Thailand | - | R | R | S | R | R | R | S | S | R | R | S |
| T4-1-2-2 | Thailand | R | S | R | S | R | R | R | R | R | R | R | S |
| T4-1-6-2 | Thailand | S | S | S | S | R | R | R | R | R | S | R | S |
| T4-1-8-1 | Thailand | S | R | R | S | R | R | R | R | R | R | R | S |
| T4-2-10-1 | Thailand | R | R | S | S | R | R | R | R | S | S | R | S |
| T4-2-1-1 | Thailand | R | R | S | S | R | R | R | R | R | S | R | S |
| T4-2-11-1 | Thailand | R | R | S | S | R | R | R | R | R | S | R | S |
| T4-2-3-2 | Thailand | R | R | S | S | R | R | R | R | R | S | R | S |
| T4-2-7-1 | Thailand | R | R | S | S | R | R | R | R | R | R | R | S |
| T4-2-9-1 | Thailand | R | R | S | S | R | R | R | R | R | R | R | S |
| T4-2-9-2 | Thailand | R | R | S | S | R | R | R | R | R | S | R | S |
| T4-3-11-1 | Thailand | R | R | R | R | R | R | R | R | R | R | R | S |
| T4-3-1-2 | Thailand | R | R | S | R | R | R | R | R | R | S | R | S |
| T4-3-3-1 | Thailand | S | S | S | S | R | R | R | R | R | S | R | S |
| T4-3-5-1 | Thailand | R | R | R | R | R | R | R | R | R | R | R | S |
| T4-4-1-1 | Thailand | R | R | S | R | R | R | R | R | R | S | R | S |
| V13-2-2 | Vietnam | R | S | S | S | R | R | R | R | R | S | R | S |
| V3-7-10-1 | Vietnam | S | R | S | S | S | S | S | R | R | R | R | S |
| V3-7-14-1 | Vietnam | R | R | R | R | R | S | R | R | R | R | R | S |
| V4-1-1-1 | Vietnam | R | R | S | S | R | R | R | R | R | R | R | S |
| V4-1-12-1 | Vietnam | S | R | S | S | R | R | R | R | R | R | R | S |
| V4-1-14-1 | Vietnam | R | R | S | S | R | R | R | R | R | R | R | S |
| V4-1-2-1 | Vietnam | S | R | S | S | R | R | R | R | R | R | R | S |
| V4-1-3-1 | Vietnam | R | R | S | R | S | S | R | R | R | R | R | S |
| V4-1-5-1 | Vietnam | S | R | S | S | R | R | R | R | R | R | R | S |
| V4-1-7-1 | Vietnam | R | R | S | S | R | R | R | R | R | R | R | S |
| V4-1-8-1 | Vietnam | S | R | S | S | R | R | S | R | R | R | R | S |
| V4-1-9-1 | Vietnam | R | R | S | S | R | R | R | R | R | R | R | S |
| V4-4-16-1 | Vietnam | R | R | S | S | R | R | R | R | R | R | R | S |
| V4-4-3-1 | Vietnam | R | R | S | S | R | R | R | R | S | R | R | S |
| V4-4-44-1 | Vietnam | R | R | R | R | R | R | R | S | S | R | R | S |
| V4-4-5-1 | Vietnam | R | R | S | S | R | R | R | S | S | R | R | S |
| V4-4-6-1 | Vietnam | R | R | R | S | R | R | R | R | R | R | R | S |
| V4-4-7-1 | Vietnam | R | R | S | S | R | R | R | S | S | R | R | S |
| V4-4-73-1 | Vietnam | R | R | S | S | R | R | R | S | S | R | R | S |
| V4-4-8-1 | Vietnam | R | S | R | S | R | R | R | R | R | R | R | S |
| V4-4-85-1 | Vietnam | R | R | S | S | R | R | R | S | S | R | R | S |
| V4-4-9-1 | Vietnam | R | R | S | S | R | R | R | R | S | R | R | S |
| V4-5-1-2 | Vietnam | R | R | R | R | R | R | R | R | R | R | R | S |
| V4-6-1-1 | Vietnam | R | R | S | S | R | R | R | S | S | R | R | S |
| V5-24-1 | Vietnam | R | R | S | S | S | S | R | S | S | S | R | S |
| V6-10-1 | Vietnam | S | S | S | S | S | S | S | S | S | S | R | S |
| V6-2-2 | Vietnam | R | R | S | R | R | S | R | S | S | S | R | S |
| V6-27-1 | Vietnam | R | R | R | R | R | S | R | S | S | S | R | S |
| 2014LC-6-5-1 | China | R | R | R | S | S | S | R | S | S | R | R | S |
| 2014RH-1-1-1 | China | R | R | S | R | S | R | R | R | R | S | R | S |
| 2014RH-1-3-1 | China | R | R | S | S | S | R | R | S | S | S | R | S |
| 2014RH-1-5-1 | China | R | R | R | R | S | R | R | R | S | S | R | S |
| 2014RH-1-9-1 | China | R | R | S | R | R | R | R | R | R | S | R | S |
| 7'-7 | China | R | S | S | S | S | S | R | S | S | R | R | S |
| 9B-10 | China | R | R | S | R | S | S | R | S | S | R | R | S |
| BN-11 | China | R | R | S | S | S | R | R | S | S | R | R | S |
| BN-17 | China | R | R | S | R | R | R | R | S | R | R | R | S |
| BN-2 | China | R | R | R | R | R | R | R | S | S | S | R | S |
| BN-23 | China | R | R | S | R | R | R | R | S | S | R | R | S |
| BN-29 | China | R | R | S | R | S | R | R | S | S | S | R | S |
| BN-34 | China | R | R | S | S | R | S | R | S | S | R | R | S |
| BN-38 | China | R | R | S | S | S | R | R | S | S | S | R | S |
| BN-5 | China | R | R | R | R | S | R | R | S | S | R | R | S |
| BN-55 | China | R | R | S | R | S | R | R | S | S | S | R | S |
| CH0997 | China | R | R | R | R | S | R | R | S | S | R | R | S |
| CH0999 | China | R | R | S | R | S | R | R | S | S | S | R | S |
| CH1003 | China | R | R | R | R | R | R | R | S | S | S | R | S |
| CH1019 | China | R | R | S | S | S | S | R | S | S | R | R | S |
| CH1389 | China | R | R | S | R | S | R | R | S | S | R | R | S |
| CH1390 | China | S | R | S | R | S | R | R | S | S | R | R | S |
| CH1391 | China | R | R | S | S | R | S | R | S | S | R | R | S |
| CH1392 | China | R | R | S | R | S | S | R | S | S | S | R | S |
| CH1393 | China | R | R | S | R | S | R | R | S | S | R | R | S |
| CH1394 | China | R | R | S | R | S | S | R | S | S | R | R | S |
| CH1395 | China | R | R | S | R | S | S | R | S | S | R | R | S |
| CH1396 | China | R | R | S | S | S | S | R | S | S | R | R | S |
| CH1397 | China | R | R | R | R | R | R | R | S | S | S | R | S |
| CH1398 | China | R | R | S | R | S | S | R | S | S | R | R | S |
| CH1399 | China | R | R | R | S | R | R | R | R | S | R | R | S |
| CH1400 | China | R | R | S | R | S | R | R | S | S | R | R | S |
| CH1418 | China | R | R | S | S | S | S | R | S | S | S | R | S |
| CH1419 | China | R | S | S | R | S | R | R | S | S | S | S | S |
| CH1420 | China | R | R | S | S | S | S | R | S | S | R | R | S |
| CH1421 | China | R | R | S | S | S | S | R | S | S | R | R | S |
| CH1422 | China | R | S | S | S | S | R | R | S | S | S | R | S |
| CH1423 | China | R | R | R | R | R | R | R | S | S | R | R | S |
| CH1424 | China | R | R | S | S | S | S | R | S | S | S | R | S |
| CH1425 | China | R | R | R | S | S | R | R | S | S | S | R | S |
| CH1426 | China | R | R | S | S | S | R | R | R | R | S | R | S |
| CH1427 | China | R | S | S | S | R | R | R | S | S | S | R | S |
| CH1428 | China | R | R | S | R | S | R | R | S | S | R | S | S |
| CH1429 | China | R | R | S | R | R | R | R | S | S | S | R | S |
| CH1430 | China | R | R | S | R | S | R | S | S | S | S | R | S |
| CH1431 | China | R | R | S | R | S | S | R | S | S | R | R | S |
| CH1432 | China | R | R | S | S | S | S | R | S | S | S | R | S |
| CH1433 | China | R | R | S | R | S | S | R | S | S | R | R | S |
| CH1595 | China | R | S | S | R | R | R | R | R | R | S | R | S |
| CH1596 | China | R | R | S | R | R | R | R | S | S | S | R | S |
| CH1597 | China | R | S | S | R | R | R | R | S | S | R | R | S |
| CH1598 | China | R | S | S | R | S | S | R | S | S | R | R | S |
| CH1599 | China | R | R | S | R | S | S | R | S | S | R | R | S |
| CH1600 | China | R | S | S | R | R | R | R | R | R | R | R | S |
| CH1601 | China | R | R | S | R | R | R | R | R | R | R | R | S |
| CH1602 | China | R | R | S | R | R | R | R | R | R | R | R | S |
| CH1603 | China | R | R | S | R | S | R | R | S | S | R | R | S |
| CH1604 | China | R | S | S | R | S | R | R | S | S | R | S | S |
| CH1605 | China | R | R | R | R | R | R | R | R | R | R | R | S |
| CH1606 | China | R | R | S | R | S | R | R | S | S | R | R | S |
| CH1607 | China | R | R | S | R | S | R | R | S | S | R | R | S |
| CH1608 | China | R | R | S | R | S | R | R | S | S | R | R | S |
| CH1609 | China | R | R | S | R | S | R | R | S | S | R | R | S |
| CH1610 | China | R | R | S | R | S | S | R | R | R | R | R | S |
| CH1611 | China | R | R | S | R | S | S | R | R | R | R | R | S |
| CH1612 | China | R | S | S | R | S | S | S | R | R | R | R | S |
| CH1613 | China | R | S | S | R | S | S | R | R | R | R | R | S |
| CH1614 | China | R | R | R | R | R | S | R | R | R | R | R | S |
| CH1615 | China | R | R | R | R | S | S | R | R | R | R | R | S |
| CH1616 | China | R | R | R | R | R | S | R | R | R | R | R | S |
| CH1617 | China | R | S | S | R | S | S | R | R | R | R | R | S |
| CH1618 | China | R | R | S | S | S | S | R | R | R | R | R | S |
| CH1619 | China | R | R | S | R | R | S | R | R | R | R | R | S |
| CH1620 | China | R | R | R | R | R | S | R | R | R | R | R | S |
| CH1621 | China | R | S | S | R | S | S | R | R | R | R | R | S |
| CH1622 | China | R | S | S | R | S | S | R | R | R | R | R | S |
| CH1623 | China | R | R | S | R | S | S | R | R | R | R | R | S |
| CH1624 | China | R | S | R | R | S | S | S | R | R | R | R | S |
| CH1625 | China | R | S | S | R | S | S | R | R | R | R | R | S |
| CH1626 | China | R | R | S | R | R | R | R | R | R | R | R | S |
| CH1627 | China | R | R | S | R | R | R | R | R | R | R | R | S |
| CH1628 | China | R | R | S | R | R | R | R | R | R | R | R | S |
| CH1629 | China | R | R | S | R | S | S | R | S | S | R | R | S |
| CH1630 | China | R | R | R | R | S | S | R | R | S | R | R | S |
| CH1631 | China | S | R | S | S | R | R | R | R | S | S | R | S |
| CH1632 | China | S | R | S | S | R | R | R | R | R | S | R | S |
| CH1633 | China | S | S | S | S | S | S | R | R | R | S | R | S |
| CH1634 | China | R | R | S | S | R | R | R | R | R | S | R | S |
| CH1635 | China | S | R | S | S | R | R | R | S | S | S | R | S |
| CH1636 | China | S | R | S | R | R | R | R | R | R | R | R | S |
| CH1637 | China | S | R | R | S | R | R | R | R | R | R | R | S |
| CH1638 | China | R | R | S | S | S | S | R | S | S | R | S | S |
| CH1641 | China | R | R | S | R | R | R | R | S | S | R | R | S |
| CH1642 | China | R | R | S | R | R | S | R | R | S | R | R | S |
| CH1643 | China | R | R | S | S | S | R | S | S | S | R | R | S |
| CH1644 | China | R | R | S | R | R | R | R | S | S | R | S | S |
| CH1645 | China | R | R | S | R | R | R | R | S | S | R | R | S |
| CH1646 | China | R | R | R | R | R | R | R | R | R | R | R | S |
| CH1647 | China | R | R | S | R | R | R | R | S | S | R | R | S |
| CH1648 | China | R | R | S | R | R | S | R | R | R | R | R | S |
| CH1649 | China | R | R | S | R | S | S | R | R | R | R | R | S |
| CH1650 | China | R | S | S | R | R | S | R | S | S | R | R | S |
| CH1651 | China | R | R | S | R | S | S | R | R | R | R | R | S |
| CH1652 | China | R | R | R | R | R | R | R | R | R | R | R | S |
| CH1653 | China | R | R | S | R | R | R | R | R | S | R | R | S |
| CH1654 | China | R | R | S | R | R | R | R | R | S | R | R | S |
| CH1655 | China | R | R | S | R | R | R | R | R | R | R | R | S |
| CH1656 | China | R | R | R | R | R | R | R | R | R | R | R | S |
| CH1657 | China | S | R | S | R | R | R | R | R | R | R | R | S |
| CH1659 | China | R | R | S | R | R | R | R | R | R | R | R | S |
| CH1661 | China | R | R | R | R | S | S | R | R | R | S | R | S |
| CH1663 | China | R | R | S | S | R | S | R | R | R | S | R | S |
| CH1664 | China | R | R | S | S | S | S | R | R | R | S | R | S |
| CH1665 | China | R | R | S | S | R | R | R | S | R | R | R | S |
| CH1666 | China | R | R | S | S | R | R | R | R | R | S | R | S |
| CH1667 | China | R | R | S | S | R | R | R | R | R | S | R | S |
| CH1668 | China | S | S | S | S | R | R | R | R | R | S | R | S |
| CH1669 | China | R | R | R | S | R | R | R | R | R | S | R | S |
| CH1670 | China | R | R | S | S | R | R | R | R | R | S | R | S |
| CH1671 | China | S | R | R | R | R | R | R | R | R | S | R | S |
| CH1672 | China | R | S | R | R | R | R | R | R | R | S | R | S |
| CH1673 | China | R | R | S | S | R | R | R | R | R | S | R | S |
| CH1674 | China | R | R | S | R | R | R | R | R | R | S | R | S |
| CH1675 | China | R | R | S | S | R | R | R | R | R | S | R | S |
| CH1677 | China | R | R | S | S | R | R | R | R | R | S | R | S |
| CH1678 | China | S | R | S | R | R | R | R | R | R | R | R | S |
| CH1679 | China | R | R | R | S | R | R | R | R | R | S | R | S |
| CH1680 | China | R | R | S | S | R | R | R | R | R | R | R | S |
| CH1681 | China | R | R | S | S | R | R | R | R | R | S | R | S |
| CH1682 | China | R | R | S | R | R | R | R | R | R | R | R | S |
| CH1683 | China | R | S | S | R | R | R | R | R | R | R | R | S |
| CH1684 | China | R | S | R | R | R | R | R | R | R | R | R | S |
| CH1685 | China | R | R | S | R | R | R | R | R | R | R | R | S |
| CH1686 | China | R | S | S | R | R | R | R | R | R | R | R | S |
| CH1687 | China | R | R | S | R | R | R | R | R | R | R | R | S |
| CH1688 | China | R | R | R | R | R | R | R | R | R | R | R | S |
| CH1689 | China | R | R | S | R | R | R | R | R | R | R | R | S |
| CH1690 | China | R | R | S | R | R | R | R | R | R | R | R | S |
| CH1691 | China | R | S | S | R | R | R | R | R | R | S | R | S |
| CH1692 | China | R | S | S | S | R | R | R | S | S | S | R | S |
| CH1693 | China | S | S | S | S | R | R | R | R | R | R | R | S |
| CH1694 | China | R | R | S | R | R | R | R | R | S | R | R | S |
| CH1695 | China | R | R | S | R | S | S | R | R | R | R | R | S |
| CH1696 | China | R | R | R | R | R | S | R | R | R | R | R | S |
| CH1700 | China | S | S | S | S | R | R | R | R | R | S | R | S |
| CH1702 | China | R | R | R | R | R | R | R | R | R | S | R | S |
| CH1703 | China | S | R | S | S | R | R | R | R | R | S | R | S |
| CH1704 | China | R | R | R | R | R | R | R | R | R | R | R | S |
| CX-15 | China | R | R | S | R | R | R | R | S | S | R | R | S |
| CY-16 | China | R | S | S | S | R | R | R | R | S | S | R | S |
| CY-4 | China | R | R | R | S | R | R | R | R | R | R | R | S |
| D111 | China | R | R | S | R | R | R | R | R | R | S | R | S |
| D142 | China | S | R | S | S | S | S | R | R | R | S | R | S |
| D157 | China | R | R | S | R | R | S | R | S | S | S | R | S |
| D164 | China | R | R | S | S | R | S | S | R | S | R | R | S |
| DQ-2 | China | R | R | S | S | R | R | R | S | S | R | R | S |
| H2-10-1 | China | R | R | S | R | S | R | R | R | R | R | R | S |
| H2-12-2 | China | R | R | S | R | R | R | R | S | S | R | R | S |
| H2-13-2 | China | R | R | S | R | R | R | S | S | S | R | R | S |
| H9-1-7 | China | R | R | S | R | R | R | R | R | R | R | R | S |
| HQ1 | China | R | R | R | R | R | R | R | R | R | R | R | S |
| ML-22 | China | R | R | R | R | R | R | R | R | R | R | R | S |
| ML-31 | China | R | R | R | R | R | R | R | R | R | R | R | S |
| ML-9 | China | R | S | S | R | R | R | R | R | R | R | R | S |
| ND-1 | China | R | S | S | R | S | S | R | S | S | R | R | S |
| ND-17 | China | R | S | S | R | R | R | R | S | S | R | R | S |
| ND-21 | China | R | S | S | R | S | S | R | S | S | R | R | S |
| RK-22-3-1 | China | R | R | S | R | R | R | R | S | S | R | R | S |
| SM-1 | China | R | R | S | R | R | R | R | R | R | R | R | S |
| SM-18 | China | R | R | S | R | R | R | R | R | R | R | R | S |
| W11-9-1 | China | R | R | S | R | S | R | R | S | S | R | R | S |
| WS-6 | China | S | S | S | R | R | S | R | R | R | R | R | S |
| YJ-43 | China | R | R | S | S | R | R | R | S | S | R | R | S |
| YL-105 | China | R | R | S | S | R | R | R | S | S | S | R | S |
| YL-13 | China | R | R | S | R | S | R | R | S | S | R | R | S |
| YL-6 | China | R | R | R | S | S | R | S | S | S | S | R | S |
| R: resistant; S: susceptible; LTH (Lijiangxintuanheigu): susceptible check; "-": not inoculated. | | | | | | | | | | | | | |
